# Clinical Prognosis of Patients With Mild COVID-19 Treated With Casirivimab/Imdevimab in Japan

**DOI:** 10.7759/cureus.21882

**Published:** 2022-02-03

**Authors:** Yasuhiro Osugi, Hitoshi Iwata, Yasushi Imai, Daiki Kobayashi, Ryutaro Hirashima

**Affiliations:** 1 Community Medicine, Fujita Health University, Toyoake, JPN; 2 Community Medicine, Toyota Regional Medical Center, Toyota, JPN; 3 Internal Medicine, St. Luke's International Hospital, Tokyo, JPN

**Keywords:** casirivimab/imdevimab administration, mild covid-19, covid-19, prognosis, cassilibimab/immudebimab

## Abstract

Aim: This study aimed to report clinical courses of patients who had mild coronavirus disease 2019 (COVID-19), defined as SpO_2_ of 96 or higher, and treated with/without casirivimab/imdevimab in Japan, where mortality and number of severe patients were very limited compared to other resource-rich countries.

Methods: We conducted a retrospective cohort study in Toyota Regional Medical Center, Toyota, Japan, from August 31, 2021, to September 27, 2021. We included all patients with COVID-19 who were diagnosed at the hospital’s family medicine department. Our primary outcome was admission to the hospital due to COVID-19 and secondary outcome was mortality due to COVID-19. We compared those who received casirivimab/imdevimab and those who did not.

Results: A total of 104 patients were included, of whom 30 received casirivimab/imdevimab and 74 did not receive casirivimab/imdevimab. The mean age of the patients was 47.8 ± 15.6 (standard deviation {SD}) years, 57 (54.8%) patients were male. During a median follow-up period of 12 days (interquartile range: 10-16 days), 19 (18.3%) patients were admitted to the hospital and none died. Patients who received casirivimab/imdevimab had similar rate to admission (p = 0.87). The hazard ratio (HR) of admission tended to be lower for those who received casirivimab/imdevimab (HR: 0.76, 95% confidence interval {CI}: 0.23-2.49, p-value = 0.65), but not statistically significant compared to those who did not, after adjusting for age, gender, risk factors, including obesity.

Conclusions: Our study demonstrated that patients with COVID-19 had similar disease progression rates regardless of casirivimab/imdevimab administration.

## Introduction

One of the potentially useful treatments for coronavirus disease 2019 (COVID-19) is anti-severe acute respiratory syndrome coronavirus 2 (SARS-CoV-2) antibody products. Previous randomized controlled study demonstrated that early administration of the monoclonal antibodies casirivimab/imdevimab in combination reduces mortality due to COVID-19 and admission to medical facilities in patients with COVID-19 [1]. Following the United States and other COVID-19 pandemic countries, Japan's Ministry of Health, Labour and Welfare (MHLW) has approved Regeneron's casirivimab and imdevimab antibody cocktail to treat patients with mild-to-moderate COVID-19 [2].

However, it was said that there may be racial or social habits differences in resistance to COVID-19. In fact, the number of COVID-19 patients was widely different in Asian countries, especially in Japan, and other Western countries, regardless of viral variants, although frequencies and capacities of the tests for SARS-CoV-2 virus would be different across countries [3]. In addition, mortality and number of patients in severe conditions are also dramatically different, although treatments for COVID-19 are now almost consistent globally. These differences are said to come from racial or social habits differences. In this study, we reported clinical courses of patients who had mild COVID-19 and were treated with/without casirivimab/imdevimab in Japan, where mortality and number of severe patients were very limited compared to other resource-rich countries.

## Materials and methods

Patients and ethical approval

We conducted a retrospective cohort study in Toyota Regional Medical Center, Toyota, Japan, from August 31, 2021, to September 27, 2021. We included all patients with COVID-19 who were diagnosed at the hospital’s family medicine department. Our primary outcome was admission to the hospital due to COVID-19 and secondary outcome was mortality due to COVID-19. We compared those who received casirivimab/imdevimab and those who did not. The ethical committee of the hospital has approved this study (approval number: 2021-kenrin13).

Casirivimab/imdevimab administration and outcomes

Single-dose of casirivimab (600 mg)/imdevimab (600 mg) was administered at the earliest available opportunity after the diagnosis based on each physician’s decision. However, patients who received casirivimab/imdevimab had to have at least one risk factor for mortality due to COVID-19, such as cardiovascular disease, chronic lung disease, diabetes, chronic kidney disease, chronic liver disease, immuno-compromised status, smoking (less than 20 packs-years), or obesity.

Our primary outcome was admission to the hospital due to COVID-19. Whether to admit to the hospital was judged based on each physician’s decision. However, patients who required oxygen therapy due to hypoxia (SpO_2_ of 93 or less) or had severe conditions, such as shock, were usually decided to be admitted.

Covariates and statistical analysis

We obtained patient information, including demographic features and known risk factors for mortality due to COVID-19 [4-7]. First, we compared baseline characteristics between patients who received casirivimab/imdevimab and those who did not receive by Fisher's exact test or t-test. Then, we compared admission to the hospital between the two patient groups by Kaplan-Meier curves and the log-rank test. We next analyzed outcomes by a multivariate Cox proportional hazards model after adjusting for covariates. All analyses were performed by Stata 14.0 (College Station, TX: StataCorp LLC).

## Results

A total of 104 patients were included, of whom 30 received casirivimab/imdevimab and 74 did not receive casirivimab/imdevimab. The mean age of the patients was 47.8 ± 15.6 (standard deviation {SD}) years, 57 (54.8%) patients were male. Table 1 shows the comparison of baseline demographic characteristics of patients who received casirivimab/imdevimab and those who did not receive it. Those who received casirivimab/imdevimab were more likely to be older (p < 0.01) and to be obese (p < 0.01). In terms of risk factors for mortality due to COVID-19, those who received casirivimab/imdevimab had similar factors compared to those who did not.

**Table 1:** Comparison of baseline demographic characteristics of patients who received casirivimab/imdevimab and those who did not receive it. *P-value < 0.05.

Characteristic	Received casirivimab/imdevimab (n = 30)	Not received casirivimab/imdevimab (n = 74)	Total (n = 104)	p-Value
Age, years (SD)	54.5 (14.6)*	45.1 (15.3)*	47.8 (15.6)*	< 0.01*
Male, n (%)	18 (60.0)	39 (52.7)	57 (54.8)	0.52
Admission to the hospital, n (%)	4 (13.3)	15 (20.3)	19 (18.3)	0.58
Body mass index, kg/m^2^ (SD)	29.6 (6.1)*	25.3 (5.6)*	25.3 (5.6)*	< 0.01*
Risk factors				0.45
Cardiovascular disease, n (%)	6 (20.0)	12 (16.2)	18 (17.3)	0.78
Chronic lung disease, n (%)	6 (20.0)	13 (17.6)	19 (18.3)	0.78
Diabetes, n (%)	3 (10.0)	12 (16.2)	15 (14.4)	0.55
Chronic kidney disease, n (%)	0 (0.0)	2 (2.7)	2 (1.9)	1.00
Chronic liver disease, n (%)	1 (3.3)	2 (2.7)	3 (2.9)	1.00
Immuno-compromised, n (%)	1 (3.3)	1 (1.4)	2 (1.9)	0.50
Smoking (less than 20 packs-years), n (%)	4 (13.3)	10 (13.5)	14 (13.5)	1.00

**Figure 1:**
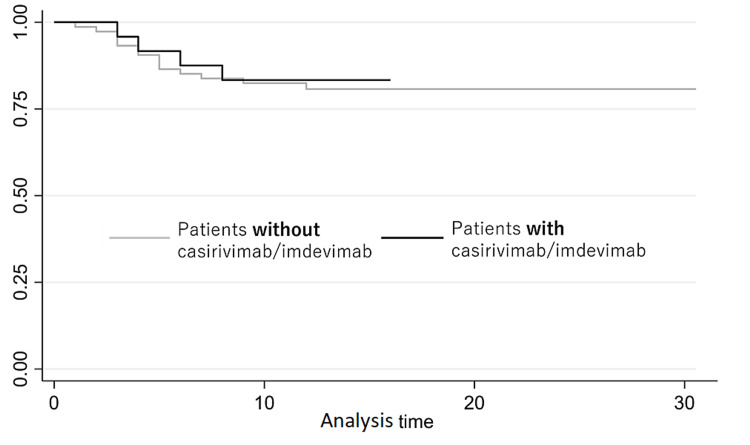
Kaplan-Meier survival curves for patients who received casirivimab/imdevimab and those who did not receive it.

Figure 1 compares each component of the Charlson comorbidity index between those who received antiplatelet drugs and those who did not. During a median follow-up period of 12 days (interquartile range: 10-16 days), 19 (18.3%) patients were admitted to the hospital and none died. Figure 1 shows the Kaplan-Meier curves for patients who received and who did not receive casirivimab/imdevimab. Patients who received casirivimab/imdevimab had a similar rate to admission (p = 0.87). The hazard ratio (HR) of admission tended to be lower for those who received casirivimab/imdevimab (HR: 0.76; 95% confidence interval {CI}: 0.23-2.49, p-value = 0.65), but not statistically significant compared to those who did not, after adjusting for age, gender, risk factors, including obesity (BMI of 25 or greater).

## Discussion

Our study demonstrated that patients with COVID-19 had similar disease progression rates regardless of casirivimab/imdevimab administration, which was inconsistent with recent randomized controlled trials [1,8,9]. Our findings may not deny the efficacy of casirivimab/imdevimab, which was shown in previous studies. There may be the following reasons why our findings were different from previous ones. First, there may be large differences in patients’ baseline characteristics, especially for age and body mass index, between those who had received casirivimab/imdevimab and those who had not. Although we adjusted these factors in multivariable Cox proportional hazard model, we may not be able to fully remove the selection bias due to the difference in patients’ characteristics. Second, unmeasured covariates may affect the findings, because the use of casirivimab/imdevimab totally depended on each physician’s decision. The physician may predict worsened clinical course based on unmeasured clinical information, deciding to administer casirivimab/imdevimab. Finally, treatment efficacy with casirivimab/imdevimab only for mild COVID-19 patients or Japanese COVID-19 patients, or both, may be limited. Previous studies included not only mild patients but also moderate patients. In addition, as we discussed in the introduction, racial or social habits differences may be associated with limited efficacy of casirivimab/imdevimab.

Although our study showed limited efficacy of casirivimab/imdevimab to Japanese patients with mild COVID-19, it was still meaningful. Currently, there are concerns that the Omicron variant may have a significant reduction in neutralization by monoclonal antibody [10,11]. Our study population mainly consisted of patients with Delta variant. Considering both the concerns about Omicron variant's resistance against monoclonal antibody and our findings that monoclonal antibody may have limited efficacy for Japanese patients with mild COVID-19, casirivimab/imdevimab may have limited efficacy for current Japanese patients with Omicron variant and certain risk factors, such as old age and obesity. Therefore, this study may be useful to caution the use of casirivimab/imdevimab for patients with Omicron variant.

Our study has several limitations. First, administration of casirivimab/imdevimab was decided by each physician, not randomly, there could be selection bias. Second, because Japan has very low COVID-19 mortality and we focused on mild patients, we could not assess hard outcomes, such as mortality or intubation rates. However, this study is still useful because the majority of COVID-19 patients had mild symptoms. Finally, our study did not have the data about vaccination rate against COVID-19. If there was a difference in the vaccination rates between those who received casirivimab/imdevimab and those who did not, this difference may bias the results.

## Conclusions

Patients with mild COVID-19 had similar disease progression rates regardless of casirivimab/imdevimab administration in Japan, where mortality and the number of severe patients were limited. This may be due to the difference in patients' characteristics between those who received casirivimab/imdevimab and those who did not. From the findings, those who were older or had obesity may have limited efficacy of casirivimab/imdevimab. These findings may be useful to caution for the use of casirivimab/imdevimab in the patients with the Omicron variant and who have specific risk factors.
